# Mortality, cause of death and risk factors in patients with alcohol use disorder alone or poly-substance use disorders: a 19-year prospective cohort study

**DOI:** 10.1186/s12888-019-2077-8

**Published:** 2019-03-28

**Authors:** Arne Jan Hjemsæter, Jørgen G. Bramness, Robert Drake, Ivar Skeie, Bent Monsbakken, Jūratė Šaltytė Benth, Anne S. Landheim

**Affiliations:** 1 Norwegian National Advisory Unit on Concurrent Substance Abuse and Mental Health Disorders, Innlandet Hospital Trust, Ottestad, Norway; 2 SERAF, Norwegian Centre for Addiction Research, University of Oslo, Oslo, Norway; 3 Institute of Clinical Medicine, University of Tromsø, Tromsø, Norway; 4 IPS Employment Center, Lebanon, USA; 5 Innlandet Hospital Trust, Gjøvik, Norway; 6 Institute of Clinical Medicine, Campus Ahus, University of Oslo, Oslo, Norway; 7 Health Services Research Unit, Akershus University Hospital, Oslo, Norway; 8 Department of Public Health, Inland Norway University of Applied Sciences, Elverum, Norway

**Keywords:** Mortality, Risk factors, Substance use disorders, Alcohol, Poly-substance use: psychiatric co-morbidity, Cause of death, Longitudinal

## Abstract

**Background:**

This study investigated cause of death, mortality rates and explored if baseline characteristics were associated with risk of death in patients with alcohol use disorder alone or poly-substance use disorders.

**Methods:**

This was a prospective, longitudinal study of patients followed for 19 years after entering specialized treatment for substance use disorders. At baseline 291 patients (mean age 38.3 years, standard deviation 11.4 years, 72% male) with high psychiatric co-morbidity were recruited; 130 (45%) had lifetime alcohol use disorder alone, while 161 (55%) had poly-substance use disorders. Time and causes of death were gathered from the Norwegian Cause of Death Registry. Lifetime psychiatric symptom disorders and substance use disorders at baseline were measured with The Composite International Diagnostic Interview and personality disorders at baseline were measured with The Millon Clinical Multiaxial Inventory II.

**Results:**

Patients with alcohol use disorder alone more often died from somatic diseases (58% versus 28%, *p* = 0.004) and more seldom from overdoses (9% versus 33%, *p* = 0.002) compared with patients with poly-substance use disorders. The crude mortality rate per 100 person year was 2.2 (95% confidence interval: 1.8–2.7), and the standardized mortality rate was 3.8 (95% confidence interval: 3.2–4.6) in the entire cohort during 19 years after entering treatment. Having lifetime affective disorder at baseline was associated with lower risk of death (Hazard Ratio 0.58, 95% confidence interval: 0.37–0.91). Older age was associated to increased risk of death among men (*p* < 0.001) and non-significantly among patients with poly-substance use (*p* = 0.057). The difference in association between age and risk of death was significantly different between men and women (*p* = 0.011) and patients with alcohol use disorder alone and poly-substance use disorders (*p* = 0.041).

**Conclusions:**

Patients with alcohol use disorder alone died more often from somatic disease than patients with poly-substance use disorders, and all subgroups of patients had an increased risk of death compared with the general population. Men with long-lasting substance use disorders are a priority group to approach with directed preventive measures for somatic health before they reach 50 years of age.

## Background

All mental disorders are associated with an increased risk of premature mortality compared with the general population, and substance use disorder (SUD) has the highest mortality rates [1, 2]. A report on drug-related mortality in nine European countries found that the crude mortality rate (CMR) per 100 person years (PY) was 1.42 (95% confidence interval [CI]: 1.37–1.47) [3]. A global review on mortality from illicit use of opiates reported the CMR per 100 PY to be 2.09 (95% CI: 1.93–2.26), with overdose being the most common cause of death, and the standardized mortality ratio (SMR) was 14.66 (95% CI: 12.82–16.50) [4]. SMR compares the deaths observed to the number of deaths expected based on age and sex at a given time in the general population. Opiate overdose mortality has increased dramatically in several parts of the world during the latest decades [5–7]. A review of alcohol use showed that the relative risk of death in clinical samples with alcohol use disorder (AUD) was 3.38 (95% CI: 2.98–3.84) for men and 4.57 (95% CI: 3.86–5.42) for women compared with the general population [8]. Mortality rates in patients treated for the first time for AUD are lower than in other clinical populations of patients with AUD [9, 10], indicating that increased severity of AUD increases risk of death. Patients with AUD have increased risk of death from cardiovascular, digestive, respiratory and endocrine diseases, cancer, mental disorders, suicide, and injuries [11]. There is less research on mortality related to the use of substances other than opiates and alcohol, but a national register study of all patients receiving treatment for SUDs in Denmark found SMRs for principal cannabis use of 4.9 (95% CI: 4.2–5.8), principal cocaine use of 6.4 (CI: 3.9–10.0), and principal amphetamine use of 6.0 (CI: 4.2–8.3) [12]. Research on mortality associated with SUDs typically reports that men have a higher CMR but lower SMR than women, younger people have a lower CMR but higher SMR than older people, and SMRs are higher in clinical cohorts than in more general populations [3, 4, 13–15]. Both CMR and SMR convey information necessary to understanding mortality, and the described differences in these rates are related to life expectancy in subgroups of the general population.

There are high levels of co-morbidity between SUDs and other mental disorders in both general and clinical populations [16, 17]. Mortality rates in patients with mental disorders are higher among those with a co-morbid SUD than those without an SUD [18, 19]. Few longitudinal studies of the mortality of patients with SUDs focus on the effect of co-morbid mental disorders. A study of patients in treatment programmes for SUDs in England showed that poly-substance use and anxiety increased the risk of death [20]. In a cohort of patients with SUDs in Sweden in which 59% were poly-substance abusers at first admission, regular use of opiates or barbiturates and neurosis increased the risk of premature death, while cannabis and psychosis decreased the risk [21]. There is a lack of research on mortality in heterogeneous clinical cohorts or comparing mortality in persons with AUD alone with persons with poly-SUDs.

To improve treatment and preventive measures to reduce mortality, we need nuanced knowledge of the risk of death, and from what causes and at which times, in various subgroups of people with SUDs [4, 11]. In the present study we examined mortality over 19 years in a heterogeneous cohort of patients with AUD alone or poly-SUDs and high psychiatric co-morbidity. Our aims were to investigate: (1) characteristics of deceased patients and principal causes of death; (2) CMRs and SMRs after entering treatment for SUDs; and (3) explore whether any baseline characteristics are associated with the risk of death in patients with AUD alone and those with poly-SUDs.

## Methods

### Design

The present design was a prospective, longitudinal study of a naturalistic cohort of patients with SUDs followed for 19 years after entering specialized treatment for SUDs in public facilities in two counties in eastern Norway in 1997 and 1998. The cross sectional study at baseline aimed to explore the prevalence of mental disorders in the cohort. Prospective follow-up studies in 2004 and 2015/2016 aimed to explore prognosis and risk-factors for different substance use and mental health outcomes, and mortality.

### Sample

At baseline, 291 patients from three outpatient (42%) and six inpatient (58%) units for specialized treatment of SUDs, owned or founded by the county councils of Hedmark or Oppland, were recruited. The inpatient units had various treatment profiles according to only alcohol use disorder (AUD) versus other SUDs or both, gender divided treatment, being a therapeutic community, and estimated length of treatment varied from 6 weeks to 18 months. The patients in the inpatient units had been referred from outpatient units. To be included in the study, the subjects had to remain in the inpatient unit for at least two weeks, or complete at least three consultations in the outpatient units. Sampling, subjects and methods at baseline have been described more extensively elsewhere [22, 23]. Compared with a national sample (*n* = 5000) of patients in facilities for specialized treatment for SUD in Norway in the same period, our sample was skewed toward having older patients with a longer duration of SUD and a higher frequency of AUD [22].

### Measurements

Norwegian Cause of Death Registry: National identity numbers for all patients (*n* = 291) were merged with data from the Norwegian Cause of Death Registry. This provided dates of death until 31 December 2016, and causes of death with medical diagnoses from death certificates (one principal cause-of-death diagnosis, and up to seven underlying diagnoses, based on the World Health Organization International Classification of Diseases-10 [ICD-10] [24]). The cause of death of deceased patients was categorized into ‘overdose’, ‘somatic’, ‘traumatic’, and ‘other’ from the principal cause-of-death diagnoses. The reference population for calculating SMRs included all residents in Norway aged 25–84 in 2008. Annual number of all-cause deaths in gender-stratified five-year age groups were obtained from the Norwegian Institute of Public Health [25], and annual population figures in the age groups 25–84 in 2008 were obtained from Statistics Norway [26].

### Baseline

The Composite International Diagnostic Interview (CIDI), Norwegian computer version - a structured personal psychiatric interview based on Diagnostic and Statistical Manual of Mental Disorders, 4th Edition (DSM-IV) criteria - and the corresponding non-hierarchical diagnoses in the ICD-10 [27] were used for lifetime psychiatric symptom disorders and SUDs. Four patients did not complete all sections on affective disorders in the CIDI, and eight did not complete all sections on anxiety disorders. The Millon Clinical Multiaxial Inventory II (MCMI-II) - a self-report psychiatric diagnostic (DSM-III-R) inventory [28] - was used to measure current personality disorders. Personality disorder diagnoses were assigned using a base-rate score of 85 or higher. The MCMI-II was not completed by 27 patients. The Norwegian National Client Assessment form [29], was used to provide information on socio-demographics and treatment history.

### Statistical analysis

Differences between participants with AUD alone and those with poly-SUDs at baseline, between living and deceased participants and between deceased participants with AUD alone and deceased participants with poly-SUDs at baseline were assessed using a χ^2^-test and independent samples t test. Separate psychiatric disorders were clustered into lifetime affective disorders (bipolar disorder, major depressive disorder and dysthymia), lifetime anxiety disorders (social phobia, agoraphobia, panic disorder, specific phobia, obsessive compulsive disorder, and post-traumatic stress disorder), and personality disorder (all personality disorders in MCMI-II). CMR was calculated as the number of all-cause deaths per 100 PY, and subgroups were compared by incidence rate ratios (IRRs). SMRs were calculated for all-cause deaths in the entire cohort, and by sex and age groups based on national death rates by sex and age (five-year cohorts) in the middle of the study period (year 2008). All rates and ratios were reported with 95% CIs. Data were described by means and SDs or frequencies and percentages, as appropriate.

Bivariate and multiple Cox regression models assessing factors associated with mortality were estimated for participants with complete data collection (*n* = 254 (37 participants were excluded from these models because of missing data on CIDI and/or MCMI-II)). To perform the analysis stratified by having AUD alone or poly-SUDs at baseline, the multiple Cox regression model contained interactions between AUD alone or poly-SUDs and the other covariates. Interactions between age and sex, age and affective disorder, age and first onset of an SUD before the age of 18 years, and sex and first onset of an SUD before the age of 18 years were also assessed. The model was reduced by applying Akaike’s Information Criterion (AIC); covariates and interactions between covariates were omitted if the AIC decreased. Models also contained a random intercept for the treatment unit, adjusting for possible within-unit correlations. The proportional hazards assumption was tested and satisfied for all Cox models. The results are presented as hazard ratios (HRs) and CIs. For statistical analysis, STATA 15 (StataCorp LLC) and SAS version 9.4 were employed.

## Results

At baseline the cohort (*n* = 291) had a mean age of 38.3 years (standard deviation [SD] 11.4 years) and 72% were male (Table 1). The baseline prevalence of lifetime anxiety disorder was 82%, 63% had a lifetime affective disorder, and the mean number of lifetime psychiatric symptom disorders (not including SUDs) was 3.6. The mean number of personality disorders at baseline was 2.7, while 28% had no personality disorder.

**Table 1:** Cohort (*n* = 291) descriptions and bivariate comparisons of baseline characteristics in participants with AUD alone compared to those with poly-SUDs at baseline, living and deceased participants at the 19 year follow-up study, and deceased with AUD alone to deceased with poly-SUDs at baseline

Baseline characteristics		Total (*n* = 291)	AUD alone (*n* = 130)	Poly-SUDs (*n* = 161)	*p* ^a^	Living (*n* = 189)	Deceased total cohort (*n* = 102)	*p* ^b^	Deceased AUD only (*n* = 66)	Deceased poly-SUDs (*n* = 36)	*p* ^c^
*Demographics*											
Sex, male	*n* (%)	208 (72)	101 (78)	107 (67)	0.035^d^	126 (67)	*82 (80)*	0.013^d^	57 (86)	25 (69)	0.040^d^
Age, years at baseline	mean (SD)	38.3 (11.4)	45.8 (9.86)	32.3 (8.67)	< 0.001^e^	35.1 (10.2)	*44.3 (11.1)*	< 0.001^e^	48.6 (9.4)	36.5 (9.8)	< 0.001^e^
Married or cohabitant (*n* = 288)	*n* (%)	98 (34)	55 (42)	43 (27)	0.007^d^	61 (33)	*37 (37)*	0.493^d^	25 (38)	12 (34)	0.721^d^
Upper secondary school (*n* = 285)	*n* (%)	139 (48)	75 (59)	64 (41)	0.003^d^	86 (47)	*53 (52)*	0.421^d^	36 (55)	17 (47)	0.479^d^
Fulltime ordinary work (*n* = 287)	*n* (%)	61 (21)	39 (30)	22 (14)	0.001^d^	42 (23)	*19 (19)*	0.456^d^	16 (24)	3 (9)	0.055^d^
*Mental health Diagnoses CIDI lifetime*											
Affective disorder (*n* = 287)	*n* (%)	182 (63)	75 (58)	107 (68)	0.067^d^	129 (70)	53 (52)	0.003^d^	27 (41)	26 (72)	0.002^d^
Anxiety disorder (*n* = 283)	*n* (%)	233 (82)	97 (76)	136 (88)	0.009^d^	154 (84)	79 (80)	0.412^d^	48 (73)	31 (94)	0.013^d^
Number of psychiatric symptom disorders (not SUD)	mean (SD)	3.6 (2.6)	3.2 (2.5)	3.9 (2.6)	0.029^e^	3.7 (2.6)	3.3 (2.6)	0.201^e^	2.8 (2.5)	4.2 (2.5)	0.012^e^
*Personality disorder MCMI-II*											
No personality disorder (*n* = 264)	*n* (%)	74 (28)	44 (36)	30 (21)	0.006^d^	47 (27)	27 (30)	0.608^d^	21 (36)	6 (19)	0.110^d^
Number of personality disorders (*n* = 264)	mean (SD)	2.7 (2.7)	2.4 (2.9)	3.0 (2.6)	0.107^e^	2.7 (2.7)	2.7 (2.8)	0.992^e^	2.6 (2.9)	3.0 (2.7)	0.471 ^e^
*Substance use disorders*											
Number of SUDs	mean (SD)	2.5 (2.0)	1.00 (0)	3.6 (2.0)	^f^	2.7 (2.0)	2.1 (1.8)	0.011^e^	1.00 (0)	4.0 (2.0)	^f^
Onset SUD before 18 years	*n* (%)	133 (46)	24 (18.5)	109 (68)	< 0.001^d^	97 (51)	36 (35)	0.009^d^	13 (20)	23 (64)	< 0.001^d^
Age onset SUD	mean (SD)	22.6 (10.7)	28.9 (11.4)	17.6 (6.7)	< 0.001^e^	20.8 (9.1)	26.0 (12.8)	< 0.001^e^	29.8 (12.02)	19.1 (10.14)	< 0.001^e^
AUD alone (vs. poly-SUDs)	*n* (%)	130 (45)	^f^	^f^	^f^	64 (34)	66 (65)	< 0.001^d^	^f^	^f^	^f^

^a^*p*-value baseline characteristics in participants with AUD alone compared to those with poly-SUDs at baseline

^b^*p*-value living compared to deceased participants at the 19 year follow-up study

^c^*p*-value deceased with only AUD at baseline compared to deceased with poly-SUDs at baseline

^d^χ^2^-test

^e^Independent samples t test

^f^Not relevant

Among the recruited patients, 45% had lifetime AUD alone, while 55% had poly-SUDs. This latter group had an average of 3.6 lifetime SUDs (the term ‘poly-SUDs’ is not entirely accurate, as 18 patients had only one other lifetime SUD besides AUD: six had only opiate, six had only cannabis, four had only sedative, and two had only stimulant use disorder). Patients with AUD alone was more often men, older, more often married or cohabitant, more often had completed upper secondary school or higher, more often had fulltime ordinary work, more seldom had lifetime anxiety disorders, had fewer lifetime psychiatric symptom disorders (not including SUDs), more seldom had personality disorders, more seldom had onset of first SUD before age 18 years of age and were older at onset first SUD compared with patients with poly-SUDs. Among the patients with poly-SUDs, 53% had lifetime opioid dependence, 49% lifetime stimulant dependence, 50% lifetime sedative, hypnotic, or anxiolytic dependence, 41% lifetime cannabis dependence, and 65% lifetime alcohol dependence.

### Characteristics of deceased patients and principal causes of death

Among the 291 patients entering treatment, 102 (35%) were deceased approximately 19 years later (mean age at death 54 years, SD 12 years), comprising 20 women (24%, mean age at death 48 years, SD 11 years) and 82 men (39%, mean age at death 56 years, SD 11 years). As displayed in Table 1, the deceased were more often men, older, more seldom had lifetime affective disorders, had fewer SUDs, more seldom had onset of first SUD before age 18 years of age, were older at onset first SUD and more often had AUD alone compared with living patients.

Among the patients with AUD alone, 51% were deceased (mean age at death 59 years, SD 11 years), and 36 (22%) of the patients with poly-SUDs were deceased (mean age at death 47 years, SD 11 years). Bivariate comparisons showed that the deceased with AUD only were more often males, older, less likely to have lifetime affective disorder, less likely to have lifetime anxiety disorder, had fewer lifetime psychiatric symptom disorders (not including SUDs), less likely to have experienced the first onset of an SUD before 18 years of age and were older at first onset SUD compared with deceased patients with poly-SUDs.

In the total cohort, 48 (47%) had somatic disease, 12 (12%) traumatic death, and 18 (18%) overdose as the principal cause of death (Table 2). More patients with AUD alone died of somatic diseases (58% versus 28%, *p* = 0.004) and fewer of overdose (9% versus 33%, *p* = 0.002) compared with participants with poly-SUDs at baseline.

**Table 2:** Principal cause of death retrieved from the Norwegian Cause of Death Registry over 19 years after entering treatment for SUDs for the deceased (*n* = 102)

		Total cohort 102 deceased	AUD alone 66 deceased	Poly-SUDs 36 deceased	*p* ^a^
Somatic disease	*n* (%)	48 (47)	38 (58)	10 (28)	0.004
Cancer	*n*	16	14	2	
Alcoholic liver disease (ICD-10 F70.0–F70.4)	*n*	7	4	3	
Heart/coronary disease	*n*	7	7	–	
Lung	*n*	8	6	2	
Others (only one participant per disease)	*n*	10	7	3	
Traumatic death	*n* (%)	12 (12)	7 (11)	5 (14)	0.623
Accidents	*n*	7	4	3	
Suicide	*n*	3	2	1	
Homicide	*n*	2	1	1	
Overdose	*n* (%)	18 (18)	6 (9)	12 (33)	0.002
Heroin	*n*	7	1	6	
Methadone	*n*	5	2	3	
‘Other’ opiods	*n*	1	–	1	
Alcohol	*n*	2	2	–	
Unspecified	*n*	3	1	2	
Other	*n* (%)	24 (24)	15 (23)	9 (25)	0.796
AUD (ICD-10, F10)^b^	*n*	15	11	4	
Opioid use disorder (ICD-10, F11)^b^	*n*	4	1	3	
No diagnosis	*n*	3	2	1	
Sudden death (ICD-10, R960)	*n*	2	1	1	

^a^*p*-value, χ^2^-test comparison of causes of death between deceased participants with AUD alone at baseline compared with deceased participants with poly-SUDs at baseline

^b^Registered as AUD or opioid use disorder as principal cause of death in the Norwegian Cause of Death Registry. Under optimal registering practice, most of these deaths should probably have been reported as somatic disease as principal cause of death

### CMRs and SMRs after entering treatment for SUDs

As displayed in Table 3, in the entire cohort, the CMR per 100 PY was 2.2. Men had a higher CMR than women, with an IRR of 1.8 (95% CI 1.1–3.0, *p* = 0.009), and younger patients had a lower CMR than older patients. Patients with AUD alone had a higher CMR than those with poly-SUDs, with an IRR of 2.6 (95% CI 1.7–4.1, *p* < 0.001). The SMR for the entire cohort was 3.8. Women had an SMR of 5.2, and males 3.6. The SMR for patients with AUD alone at baseline was 3.4, while patients with poly-SUDs at baseline had an SMR of 5.2. Not shown in Table 1 are patients who died between the ages of 25 and 39 years (*n* = 15), who had an SMR of 14.3 (95% CI 8.3–23.1); between 40 and 49 years (*n* = 19), who had an SMR of 6.4 (95% CI 4.0–9.8); between 50 and 59 years (*n* = 32), who had an SMR of 5.9 (95% CI 4.1–8.3); and above 60 years (*n* = 36), who had an SMR of 2.1 (95% CI 1.5–2.9).

**Table 3:** All-cause deaths, crude mortality rate (CMR) per 100 person years (PY), and standardized mortality ratio (SMR) in the cohort (*n* = 291) and subgroups over the 19 years after entering treatment for SUDs

	Deaths (%)	Mortality rate per 100 PY (95% CI)	Standardized mortality ratio (95% CI)
Total cohort	102 (35)	2.2 (1.8–2.7)	3.8 (3.2–4.6)
*Sex*			
Male	82 (39)	2.5 (2.1–3.2)	3.6 (2.9–4.5)
Female	20 (24)	1.4 (0.9–2.2)	5.2 (3.3–7.9)
*Age at baseline*			
Under 30 years	11 (16)	0.9 (0.5–1.6)	
30–39 years	25 (26)	1.6 (1.1–2.3)	
40–49 years	32 (48)	3.1 (2.2–4.4)	
Over 50 years	34 (61)	4.6 (3.3–6.4)	
*AUD alone at baseline*			
AUD alone	66 (51)	3.5 (2.8–4.5)	3.4 (2.6–4.2)
Poly-SUDs	36 (22)	1.3 (1.0–1.8)	5.2 (3.7–7.2)

### Baseline characteristics associated with risk of death in patients with AUD alone and poly-SUDs

Table 4 displays results from a Cox regression analysis assessing the association between baseline characteristics and risk of death over 19 years after entering treatment for SUDs. In bivariate models, age and AUD alone were associated with a higher risk of death, while having a lifetime affective disorder and experiencing the first onset of an SUD before the age of 18 years were associated with lower risk of death (all *p* < 0.05). In the multiple model, having a lifetime affective disorder (HR 0.58, 95% CI 0.37–0.91) was associated with lower risk of death. There were three interactions left in the AIC-reduced multiple model, between age and sex, age and first SUD onset before 18 years of age, and age and AUD alone. For each one-year increase in age, the risk of death increased by 6% in women (*p* = 0.057) and 12% in men (*p* < 0.001), with risk being overall significantly different between women and men (*p* = 0.011 for interaction). Exploring the interaction term further revealed that the risk of death is not significantly different between men and women in younger patients, but from an age of about 50 years onwards, men have significantly higher risk of death than women. In those with poly-SUDs increasing age was slightly but not significantly associated with higher risk for death, while the association was more or less stable in those with AUD alone. The risk was overall significantly different between those with AUD alone and patients with poly-SUDs (*p* = 0.042 for interaction). Patients up to about age of 35 years with AUD alone had higher risk of death than those with poly-SUDs, with no differences between groups among patients older than 35 years according to exploratory analysis of the interaction term. There were no significant differences in the association between age and risk for death among those with first SUD onset before the age of 18 years and age of 18 years or older, even though the interaction was not eliminated from the model by AIC.

**Table 4:** Cox regression analysis for risk of death over the 19 years after entering treatment for SUDs, *n* = 254 (cases with missing data for at least one covariate were excluded)

	Model 1^a^	Model 2^b^
	Bivariate HR (95% CI)	Multiple, AIC reduced HR (95% CI)
*Demographics*		
Sex, male (female – ref.)	1.50 (0.87–2.57)	1.12 (1.07–1.17)^c^ ***
Age	1.06 (1.04–1.08)***	1.06 (1.00–1.73)^d^
*Mental health*		
Affective disorder (no affective disorder – ref)	0.56 (0.37–0.87)**	0.58 (0.37–0.91)*
Anxiety disorder (no anxiety disorder – ref)	0.83 (0.49–1.41)	
Number of psychiatric symptom disorders (except SUDs)	0.95 (0.87–1.03)	
Personality disorder (no personality disorder – ref)	1.00 (0.63–1.60)	
*Substance use*		
First SUD onset before the age of 18 years (above 18 years – ref.)^c^	0.58 (0.37–0.93)*	1.01 (0.96–1.07)^c^
AUD alone (poly-SUDs – ref.)^c^	2.67 (1.67–4.24)***	1.00 (0.96–1.05)^c^

* *p* < 0.05, ** *p* < 0.01, *** *p <* 0.001

^a^Bivariate analysis of risk of death by 31 December 2016, adjusted for inter-unit differences

^b^Multiple model for risk of death by 31 December 2016, stratified by having AUD alone at baseline, adjusted for inter-unit differences. Stratification was performed by estimating the model containing interactions between all variables and AUD alone, and in addition to interactions between age and sex, age and affective disorder, age and first SUD onset before the age of 18 years, and sex and first SUD onset before the age of 18 years. The model was reduced by applying the AIC, leaving three interactions in the model: age and sex, age and first SUD onset before 18 years of age, and age and AUD alone

^c^HR for 1-year change in age

^d^HR for 1-year change in age in reference groups (female sex, first SUD onset at 18 or more years, poly-SUDs)

## Discussion

In the present cohort, patients with AUD alone died more often from somatic disease and more seldom from overdose than patients with poly-SUDs. More than 2% died annually through the 19-year observation period, and all examined subgroups of patients had an increased risk of death compared with the general population. In a multiple analysis, we found that lifetime affective disorder at baseline was associated with a decreased risk of death, while age and sex interact in predicting risk of death. Older age was associated to increased risk of death among men and non-significantly among patients with poly-substance use. The difference in association between age and risk of death was significantly different between men and women and patients with alcohol use alone and poly-substance use disorders.

As expected [4, 11], the principal causes of death in patients with AUD alone were mainly somatic diseases, and overdose was the most common principal cause of death in patients with poly-SUDs. This difference is important to acknowledge and have clinical implications because death from overdose or somatic causes requires different preventive approaches. Patients with long-lasting SUDs need better access to screening and treatment to reduce premature mortality from somatic causes, but this seems to receive less systematic efforts compared to overdose prevention. Several effective large-scale strategies for decreasing death by overdose exist; e.g. opioid maintenance treatment (OMT) [4], education about high-risk situations or combinations of substances, means of intake, and prevention with naloxone [30, 31]. For patients with opiate use disorder OMT decrease somatic morbidity [32]; however patients in OMT also need improved somatic health care to reduce premature mortality further [33]. Patients with SUDs also have other shared risk factors for premature mortality from somatic causes, which we have not addressed in this study, and will probably benefit from more indirect preventive measures for mortality, such as adequate treatment for SUDs and mental disorders, smoking cessation, exercise, and healthier nutrition.

Our findings for CMRs and SMRs by age group and sex are similar to those found by other Norwegian [33–36] and international studies [3, 4, 8] of mortality in patients with SUDs. These results are however influenced by a selection bias in that younger patients with poly-SUDs at high risk of mortality from overdose or traumatic causes were under-represented. The average age at baseline was 38 years, which means that the participants who entered this study had already survived SUDs for a long time.

Somewhat counterintuitive, we found that having a lifetime affective disorder was associated with a decreased risk of death, crudely adjusted for other factors. We have no knowledge of studies indicating that affective disorders decrease the risk of death in patients with SUDs, although psychosis in combination with cannabis use disorder has been reported to decrease the risk of death [21]. This may be an effect of selection bias as the patients with AUD alone had less lifetime affective disorders at baseline compared to those with poly-SUDs, and many of the deceased were older patients with AUD alone. Both men and women with SUDs had, as expected, an increased risk of death with increasing age, but men had a significantly higher risk of death than women from an age of 50 years onwards. This can have clinical implications for preventing premature death because we know most of these men die from somatic causes. Unlike some other studies [20, 21] we did not find mental disorders to be associated with an increased risk of death. These negative findings may also be related to sample characteristics.

### Limitations

The present study has limitations that must be considered when interpreting the results. We have a baseline selection bias in that younger patients at high risk of death from overdose or traumatic causes are under-represented. The cohort should ideally be larger and the study may be underpowered regarding the multiple Cox models, which increase the chance of type-II statistical error [37]. Also, the multiple analysis is only crudely adjusted as persons with SUDs have several relevant risk factors for premature mortality which we have not investigated, e.g. smoking, unhealthy lifestyle, and genetic factors. Our finding that psychiatric disorders do not increase the risk of mortality may be an effect of high baseline prevalence, which makes differences difficult to detect (i.e. Berkson’s fallacy [38]). SUDs and psychiatric co-morbidities at baseline may have changed for several patients and influenced their mortality over the 19-year study period. There can also be problems in measuring lifetime mental disorders at baseline in temporal proximity to substance abuse, and there can be differences between the diagnoses reached via structured diagnostic interviews, as we used, compared with those by clinical experts, who likely set fewer diagnoses [39].

The main strengths of the study were the 19-year study period, the heterogeneous clinical cohort with long-term problematic substance use, and the thorough diagnostic investigation with structured personal interviews at baseline. Despite possible limitations, this study provides useful and unique longitudinal data regarding the mortality of patients with AUD alone or poly-SUDs and may help to direct future research and treatment interventions.

## Conclusions

This study confirms and extends knowledge that patients with SUDs have substantially higher risk of death than the general population, and that type of SUD influences risk for different causes of death. Our findings suggest that more systematic and early attention to somatic health in patients with SUDs, and especially in older patients with long-lasting substance use problems, can be relevant for preventing premature mortality. Men with long-lasting SUDs are a priority group to approach with directed preventive measures on somatic health well before 50 years of age.

## Abbreviations

AIC: Akaike Information Criterion
AUD: Alcohol Use Disorder
CI: Confidence Interval
CIDI: Composite International Diagnostic Interview
CRM: Crude Mortality Rate
DSM-III-R: Diagnostic and Statistical Manual of Mental Disorders, 3rd Edition, Revised
DSM-IV: Diagnostic and Statistical Manual of Mental Disorders, 4th Edition
HR: Hazard Ratio
HSCL-25: Hopkins Symptom Checklist-25
ICD-10: International Classification of Diseases, 10th Edition
IRR: Incidence Rate Ratio
MCMI-II: Millon Clinical Multiaxial Inventory II
OMT: Opioid Maintenance Treatment
PY: Person Years
SD: Standard Deviation
SMR: Standardized Mortality Ratio
SUD: Substance Use Disorder
